# Osmostress Induces Autophosphorylation of Hog1 via a C-Terminal Regulatory Region That Is Conserved in p38α

**DOI:** 10.1371/journal.pone.0044749

**Published:** 2012-09-11

**Authors:** Inbal Maayan, Jonah Beenstock, Irit Marbach, Shira Tabachnick, Oded Livnah, David Engelberg

**Affiliations:** Department of Biological Chemistry, The Institute of Life Science, The Hebrew University of Jerusalem, Jerusalem, Israel; University of Pecs Medical School, Hungary

## Abstract

Many protein kinases require phosphorylation at their activation loop for induction of catalysis. Mitogen-activated protein kinases (MAPKs) are activated by a unique mode of phosphorylation, on neighboring Tyrosine and Threonine residues. Whereas many kinases obtain their activation via autophosphorylation, MAPKs are usually phosphorylated by specific, dedicated, MAPK kinases (MAP2Ks). Here we show however, that the yeast MAPK Hog1, known to be activated by the MAP2K Pbs2, is activated in *pbs2*Δ cells via an autophosphorylation activity that is induced by osmotic pressure. We mapped a novel domain at the Hog1 C-terminal region that inhibits this activity. Removal of this domain provides a Hog1 protein that is partially independent of MAP2K, namely, partially rescues osmostress sensitivity of *pbs2*Δ cells. We further mapped a short domain (7 amino acid residues long) that is critical for induction of autophosphorylation. Its removal abolishes autophosphorylation, but maintains Pbs2-mediated phosphorylation. This 7 amino acids stretch is conserved in the human p38α. Similar to the case of Hog1, it’s removal from p38α abolishes p38α’s autophosphorylation capability, but maintains, although reduces, its activation by MKK6. This study joins a few recent reports to suggest that, like many protein kinases, MAPKs are also regulated via induced autoactivation.

## Introduction

Phosphorylation on a particular Thr residue at their activation loop is one of the most common mechanisms that activate protein kinases [1]–[3]. In many kinases this phosphorylation is achieved by autophosphorylation, but some kinases are phosphorylated and activated by other kinases (e. g., CDKs by CAK; PKB and PKC by PDK1 [4]–[6]). Some kinases, such as Mitogen activated protein kinase kinases (MEKs, MAPKKs, or MAP2Ks), require two phosphorylation events for activation [7]. Members of the Mitogen Activated Protein Kinase (MAPK) family are further unique as their activation not only requires dual phosphorylation, but also occurs on a unique motif at the activation domain that includes a Tyr phosphoacceptor in addition to the Thr (a T-X-Y motif) [7]–[10]. MAPK’s dual phosphorylation is catalyzed by MAP2Ks and it was long accepted that if not phosphorylated on both phosphoacceptors, MAPKs are catalytically inactive [11]–[14]. Later studies suggested however that when monophosphorylated on the Thr residue MAPKs do manifest some catalysis, albeit low, and even biological activities [15]–[19]. In addition, MAPK activation via MAP2K-independent mechanisms was also reported, so far only for the mammalian MAPK p38α. All these MAP2K-independent mechanisms involve induction of an intrinsic autophosphorylation capability of p38α [20], [21]. The capability to autophosphorylate seems to exist, although hindered, in many MAPKs, as certain point mutations can uncover it and render the kinases intrinsically active [22]–[26]. Finally, members of the mammalian MAPK families ERK and p38 are spontaneously autophosphorylated when expressed in yeast [27].

In this report we show that the yeast MAPK Hog1 is also regulated by induction of its autophosphorylation activity. Furthermore, we mapped a novel domain in Hog1 that inhibits this activity and an adjacent domain that is critical for induction of autophosphorylation.

Hog1 is the yeast ortholog of the mammalian MAPKs JNK and p38. It was discovered as an osmostress-responsive kinase, but is known now to respond to various cues [28]–[30]. Hog1 can be activated by either of two, apparently redundant, signal transduction pathways; the Sln1-Ypd-Ssk1 and the Sho1 cascades. Both pathways lead to activation of a common MAP2K, Pbs2, which in turn dually phosphorylates Hog1. Active Hog1 allows adaptation and subsequently proliferation under osmostress [31], [32] by controlling many cellular activities including transcription and cell cycle [30], [33]–[36]. It seems that the critical mechanism through which Hog1 allows proliferation under osmostress is induction of glycerol biosynthesis [37].

Pbs2 is considered the only MAP2K for Hog1. On its absence therefore, Hog1 should not be phosphorylated and activated and should not support proliferation under high osmostress [28], [30], [35], [38]. Here we show however, that in *pbs2*Δ cells Hog1 is phosphorylated and activated to some degree in response to high osmotic pressure (0.8 M or 1 M NaCl). We report that this Pbs2-independent, osmostress-dependent Hog1 activation is achieved primarily via autophosphorylation. We mapped a C-terminal domain in Hog1 that restricts autoactivation. Removal of this domain increases Pbs2-independent Hog1 activity to levels that allow *pbs2*Δ cells to proliferate to some degree under osmotic pressure. We further mapped a small region (seven amino acids long) in Hog1’s C-terminal domain to be important for it’s autophosphorylation activity. This region partially overlaps with the Pbs2-interacting site, suggesting that Pbs-dependent and Pbs2-independent mechanisms of Hog1 activation use a similar domain. Finally, we found this region to be functionally conserved in the human p38α.

## Materials and Methods

### Yeast Strains and Media

The *S. cerevisiae* strains used were: JBY13 (*MAT*a, *leu2, ura3, his3, trp1, ade2, lys2, hog1*::*TRP1*; obtained from M. C. Gustin, Rice University), a *hog1Δpbs2Δ* strain (*MAT*a, *leu2, ura3, his3, trp1, ade2, lys2, hog1*::*TRP1, pbs2-*Δ*2*::*LEU2*; [22]) and YPH102 (*MATa, ura3, leu2, his3, ade2, lys2* [32]). Cultures were maintained on either YPD (1% yeast extract, 2% Bacto Peptone, 2% glucose), or on the synthetic medium YNB, or on YNB(-URA) (0.17%yeast nitrogen base without amino acid and (NH_4_)_2_SO_4_, 0.5% ammonium sulfate, 2% glucose and 40 mg/liter of the required nutrients. To induce osmotic stress YPD supplemented with 0.8 M NaCl or 1 M NaCl was used.

### Viability Assay - “Drops” Assay

Yeast cultures were grown in liquid medium, [YNB(-URA)], to logarithmic phase (OD_600_∼0.4). Five serial dilutions were created (unless mentioned otherwise, concentrations were approximately 10^7^, 10^6^, 10^5^, 10^4^ and 10^3^ cells/ml) and 5 µl from each dilution were plated on YPD plates supplemented with 0.8 M or 1 M NaCl. Plates were grown at 30^0^C for 5 days.

### Construction of Plasmids

All *HOG1* molecules were expressed from the pES86 vector as HA-tagged proteins, as described previously [39]. Native *HOG1* and various derivatives were sub-cloned between the *ADH1* promoter and terminator. The pES86 vector further harbors the *URA3* gene and the 2μ-element. C-terminal truncations of *HOG1* were obtained via a series of polymerase chain reactions. The same N-terminal primer was used in all reactions, but a different C-terminal primer, complementing the desired part of the protein and a stop codon, was used in each. Each PCR product was sub-cloned into pES86 and verified by sequence analysis. To create a *HOG1* gene in which the region encoding Y337–F343 is deleted, we applied the QuickChange® kit (STRATAGENE) using the primers 5′-gatacctggcgtgttatgatgcataagattggtggcagtgat-3′ and 5′-atcactgccaccaatcttatgcatcataacacgccaggtatc-3′. Appropriate removal of the fragment was verified by sequencing. pcDNA-p38α and pBabae-MKK6^EE^ were described previously [25].

### Preparation of Protein Lysates of Yeast Cells via the TCA Precipitation Method

Yeast cultures were grown in liquid medium YNB(-URA) to logarithmic phase (OD_600_∼0.5), precipitated by centrifugation (5 min., 2000×g) and re-suspended in YPD medium with or without NaCl. At each relevant time point a sample of 20 ml was collected and precipitated. The yeast pellet was washed with 10 ml of 20%TCA and precipitated again by centrifugation (10 min., 3400×g). The washed pellet was re-suspended in 200 µl of 20%TCA and 600 mg of glass beads were added. Cells were broken by vortexing for 8′ and the supernatants were transferred to a clean tube. The beads that were left in the tube were washed twice with 200 µl of 5%TCA, and the supernatants were combined to the same tube. The proteins were precipitated by centrifugation (10 min, 1800×g) and the pellet was re-suspended in 200 µl of Laemmli buffer and 100 µl of 1 M Tris base and boiled for 3′.

### Western Blot Analysis

Unless mentioned otherwise, 30µg of protein lysates were separated via 10% SDS-PAGE and subsequently transferred to a nitrocellulose paper. Nitrocellulose papers were incubated with the appropriate antibodies. Antibodies used were: anti-phospho-p38 (cell signaling #9211), anti HA antibodies (12CA5 monoclonal), anti-MAPKAPK2 (cell signaling #3042), anti-phospho MAPKAPK2 (cell signaling, #3007) and anti-flag (sigma, A2220).

### Mammalian Cell Culture

HEK293T cells were grown in Dulbeco’s MEM medium supplemented with 10% fetal bovine serum and antibiotics. Cells were grown at 37°C under 5% CO_2_. Cells were transfected with pcDNA and pBabe plasmids containing the cDNA of p38α and MKK6^EE^ respectively by the calcium phosphate method as described [25]. 24 hours post transfection cells were lysed with Laemmli buffer and lysates were subjected to western blot as described [25].

## Results

### Hog1 is Activated by Osmostress-induced Autophosphorylation

**Figure 1:**
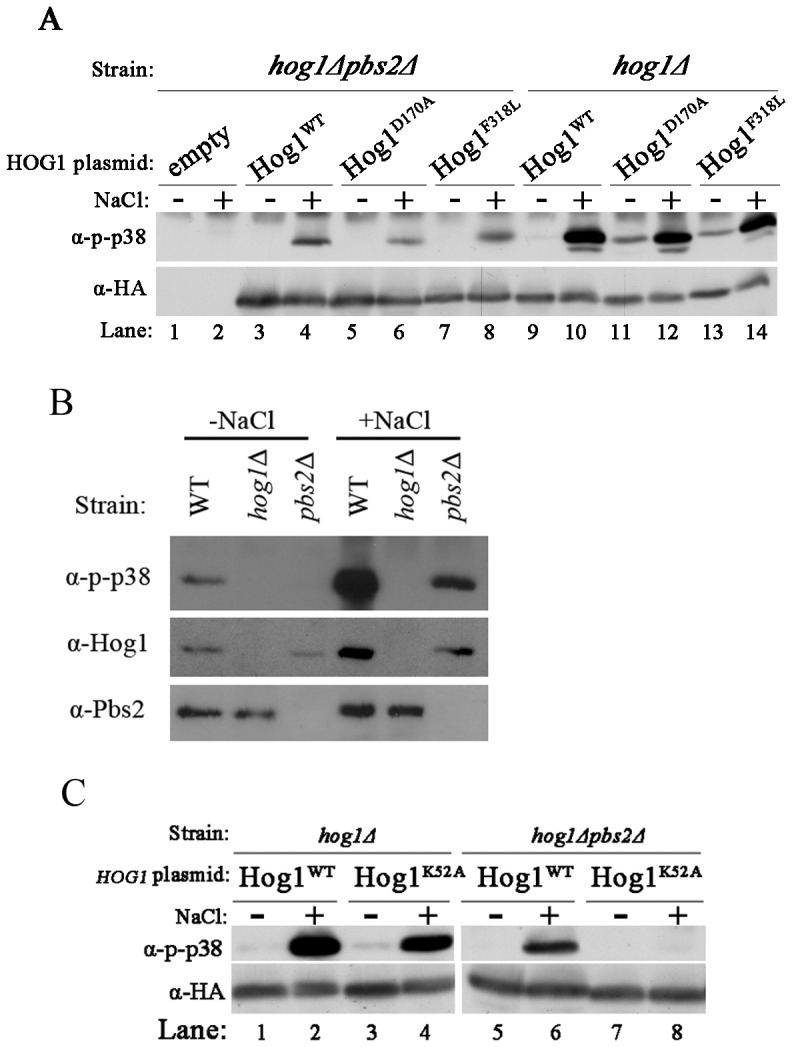
Intrinsically active Hog1 proteins as well as Hog1^WT^ are autophosphorylated in response to osmostress in the absence of Pbs2. A. Phosphorylation levels of wild type Hog1 (Hog1^WT^) and of the intrinsically active Hog1 proteins, Hog1^D170A^ and Hog1^F318L^, were tested when expressed in *hog1*Δ*pbs2*Δ cells (lanes 3–8) or in *hog1*Δ cells (lanes 9–14), before (−) and 10 minutes after (+) exposure to 1 M NaCl. *hog1*Δ*pbs2*Δ cells harboring an empty vector (lanes 1–2) served as a negative control. Cultures were grown on YNB(-URA). B. Endogenous, native Hog1^WT^ protein is phosphorylated in *pbs2*Δ cells. Phosphorylation levels of Hog1^WT^ were tested in cells of the YPH102 (WT), *hog1*Δ and *pbs2*Δ strains, grown on YPD (−NaCl) and 10 minutes after transfer to YPD media supplemented with 1 M NaCl (+NaCl). C. Kinase-dead Hog1 is not phosphorylated in *hog1*Δ*pbs2*Δ cells. Phosphorylation levels of wild type Hog1 (Hog1^WT^) and kinase-dead Hog1 (Hog1^K52A^) were tested when expressed in *hog1*Δ strain (lanes 1–4) or *hog1*Δ*pbs2*Δ strain (lanes 5–8), before (−) and 10 minutes after (+) exposure to 1 M NaCl.

In the absence of Pbs2, yeast cells cannot proliferate under high osmotic pressure because Hog1 cannot be activated. Nevertheless, *pbs2*Δ cells can proliferate under osmostress, if they express intrinsically active Hog1 molecules such as Hog1^D170A^, Hog1^F318S^ or Hog1^F318L^ [22], [39]. As these active variants do not require Pbs2, the only known Hog1 activator, the premise is that their phosphorylation status and catalytic activity are constant. Unexpectedly however, their phosphorylation levels significantly increased in cells exposed to osmotic stress, and even in *pbs2*Δ cells (Fig. 1A). More surprisingly, even Hog1^WT^, that cannot support proliferation of *pbs2*Δ cells under osmostress, was also phosphorylated in response to osmotic pressure (although to a lower level than its phosphorylation level in PBS2^+^ cells) (Fig. 1A; compare lane 4 to lane 10). To test whether the unexpected Hog1’s phosphorylation is a mere consequence of experimental manipulation, perhaps relevant only to externally introduced genes, we repeated the experiment on cells that do not harbor any foreign plasmid (Fig. 1B). This experiment showed that endogenous native Hog1 protein is phosphorylated in *pbs2*Δ cells exposed to 1 M NaCl (Fig. 1B). Thus, in addition to Pbs2-mediated activation there is a parallel activity that responds to osmotic pressure and renders a phosphorylated Hog1. This parallel activity is absolutely independent of Pbs2. In fact, phosphorylation levels of the Hog1 molecules in *pbs2*Δ cells did not change when a kinase-dead version of Pbs2 was co-expressed ([39] and data not shown), suggesting that scaffold properties of Pbs2 have no effect on this activity.

Although phosphorylation was monitored with site-specific antibodies (anti dually-phosphorylated p38), we wished to further confirm that Hog1 phosphorylation in *pbs2*Δ cells occurs on the TGY motif. We therefore expressed Hog1^T174A^, Hog1^Y176F^, or Hog1^T174A+Y176F^ in *pbs2*Δ cells. These proteins were not recognized by the anti-phspho-p38 antibodies confirming that Thr174 and Tyr176 are the phosphoacceptors in *pbs2*Δ cells (data not shown).

Hog1 phosphorylation in *pbs2*Δ cells could be explained by two possible mechanisms. One is phosphorylation by another MAP2K that partially takes over Pbs2 activity and the other is autophosphorylation. To reveal the mechanism, a kinase-dead Hog1 (carrying the K52A mutation, Hog1^K52A^) was expressed in *pbs2*Δ cells. If Hog1’s activation in these cells is achieved by autophosphorylation, the kinase-dead mutation should abolish its phosphorylation. Indeed, this protein was not phosphorylated under osmotic stress conditions in *pbs2*Δ cells (Fig. 1C, lane 8). The same kinase-dead protein was phosphorylated in cells possessing a Pbs2 protein, showing that it is a valid substrate for a relevant MAP2K (Fig. 1C, lane 4). These results strongly suggest that in the absence of Pbs2, Hog1 is not phosphorylated by another kinase.

### Hog1 Proteins Lacking the C-terminal Domain Partially Rescue *pbs2*Δ Cells from Osmostress

**Figure 2:**
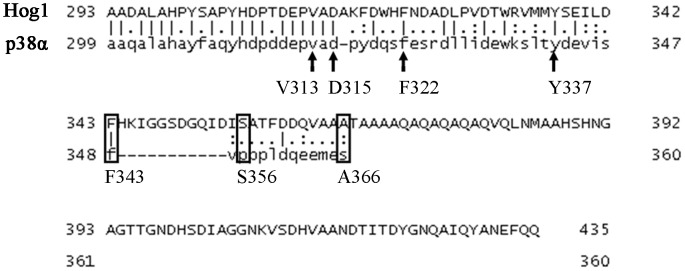
Sequence alignment of the C-terminal domains of Hog1 and p38α with indications of the last C-terminal residue of the different truncated proteins created. Rectangles: The last residue of each of the first 3 truncations that were made. Arrows: The last residue of each of the second set of truncated proteins that were created (see text for details).

**Figure 3:**
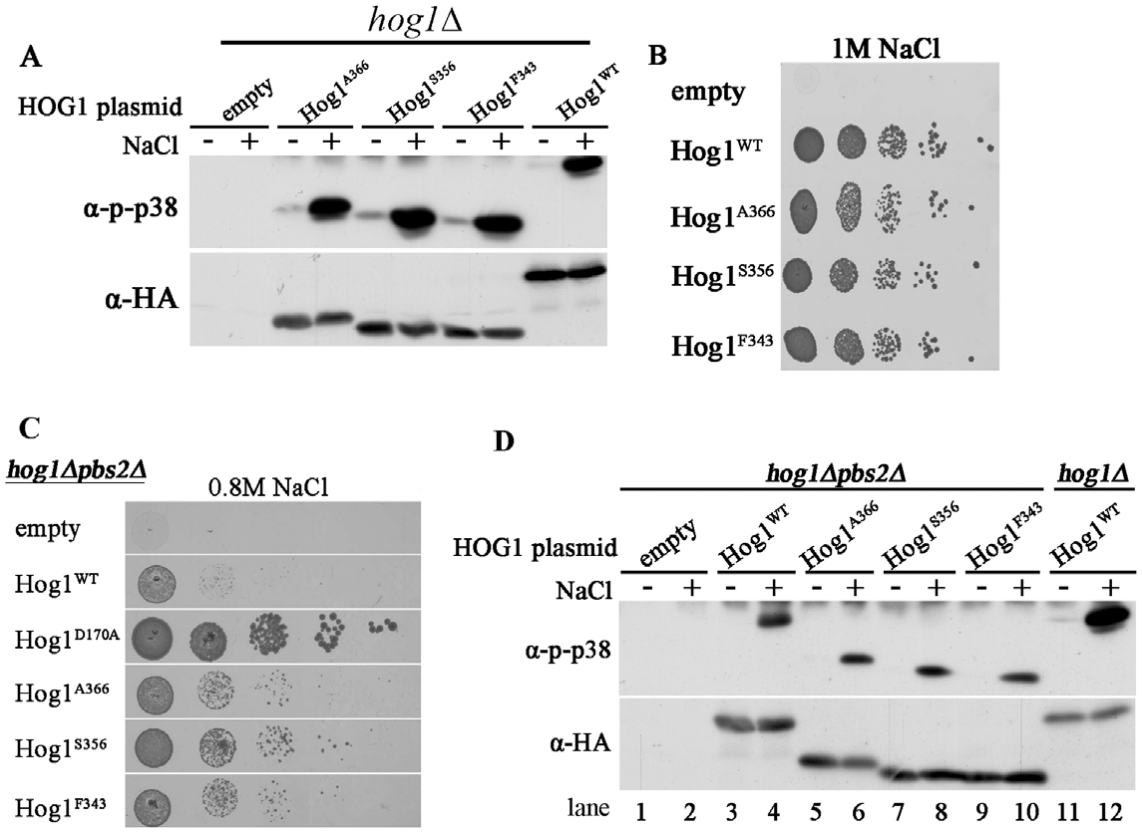
Hog1 proteins lacking the C-terminal tail are capable of partially rescuing *pbs2*Δ cells of osmostress. **A.** Phosphorylation levels of wild type Hog1 (Hog1^WT^) and of the truncated Hog1 proteins, Hog1^A366^, Hog1^S356^ and Hog1^F343^ were tested when expressed in *hog1*Δ cells, before (−) and 10 minutes after (+) exposure to 1 M NaCl. **B.** *hog1*Δ cells harboring an empty vector (negative control), or vectors expressing Hog1^WT^, or the truncated Hog1 proteins Hog1^A366^, Hog1^S356^ or Hog1^F343^ were plated in five dilutions on plates containing YPD supplemented with 1 M NaCl. All strains were also plated on medium with no NaCl and all grew equally well on these plates (not shown). **C.** *hog1*Δ*pbs2*Δ cells harboring same vectors as in panel B were plated in five dilutions on plates containing YPD supplemented with 0.8 M NaCl. Growth of cells expressing an intrinsically active Hog1 variant (Hog1^D170A^) served as a positive control. All strains were also plated on medium with no NaCl and all grew equally well on these plates (not shown). **D.** Phosphorylation levels of the indicated Hog1 proteins were tested as in panel A, but when expressed in *hog1*Δ*pbs2*Δ cells. Phosphorylation level of Hog1^WT^, expressed in *hog1*Δ strain is presented for comparison (right lanes).

The results above not only suggest autophosphorylation capability of Hog1, but further show that this activity is regulated, namely induced in response to osmotic pressure. We sought therefore identification of the domain in Hog1 that is involved in regulating this Pbs2-independent activation. We anticipated that this domain is specific to Hog1 and is absent from its mammalian ortholog p38α. This notion stems from the observation that when expressed in yeast, p38α is constitutively phosphorylated as a result of autophosphorylation [27], [40]. Namely, while Hog1’s autophosphorylation capability is kept off under optimal growth conditions, p38α’s autophosphorylation is unregulated when expressed in yeast. As Hog1 possesses a long C-terminal “tail” that is absent from p38α (Fig. 2), it could be that Hog1’s autophosphorylation is regulated by this C-terminal region. To test this notion we prepared truncated Hog1 proteins by systematically removing the most C-terminal 84–92 amino acids, creating proteins Hog1^A366^, Hog1^S356^ and Hog1^F343^ (named after their last residue) that are more similar to p38α (Fig. 2, rectangles). Truncated *HOG1* genes were sub-cloned to the 2μ-based pES86 vector downstream of the strong *ADH1* promoter. Notably, Hog1^F343^ lacks part of the major Pbs2 binding domain (PBD-2 domain) that was mapped by pull down assays to W320-D350 [41]. Hog1 has another, less efficient, Pbs2-binding domain (common docking, CD, domain) residing in S302-A316 [41]. The CD domain remains intact in all three truncated proteins. When expressed in *hog1*Δ cells the truncated proteins were spontaneously phosphorylated to some degree (i.e., even in cells that were not exposed to osmotic stress; Fig. 3A, lanes 3,5,7), reminiscent of the phosphorylation levels of the intrinsically active Hog1 mutants ([22], [39]; see also Fig. 1). Phosphorylation levels were augmented following exposure to osmostress, similar to the Hog1^WT^ (Fig. 3A), suggesting that Pbs2 efficiently phosphorylates them. Importantly, all truncated proteins were biologically active as manifested by their ability to rescue *hog1*Δ cells from osmostress just as Hog1^WT^ (Fig. 3B). It seems therefore that the 92 C-terminal amino acids (H344–Q435) are not critical for Hog1’s activity and may play in fact some inhibitory role. Indeed, the truncated Hog1 proteins partially rescued *pbs2*Δ cells (Fig. 3C), supporting the idea that they acquired some intrinsic pbs2-independent activity. Puzzlingly, phosphorylation levels of the truncated proteins were not significantly different (in fact slightly lower) than those of Hog1^WT^ that cannot rescue *pbs2*Δ cells efficiently (Fig. 3D).

Rescuing *pbs2*Δ cells by the truncated proteins, that are activated solely by autophosphorylation, may suggest that the autoactivation mechanism is of biological significance.

**Figure 4:**
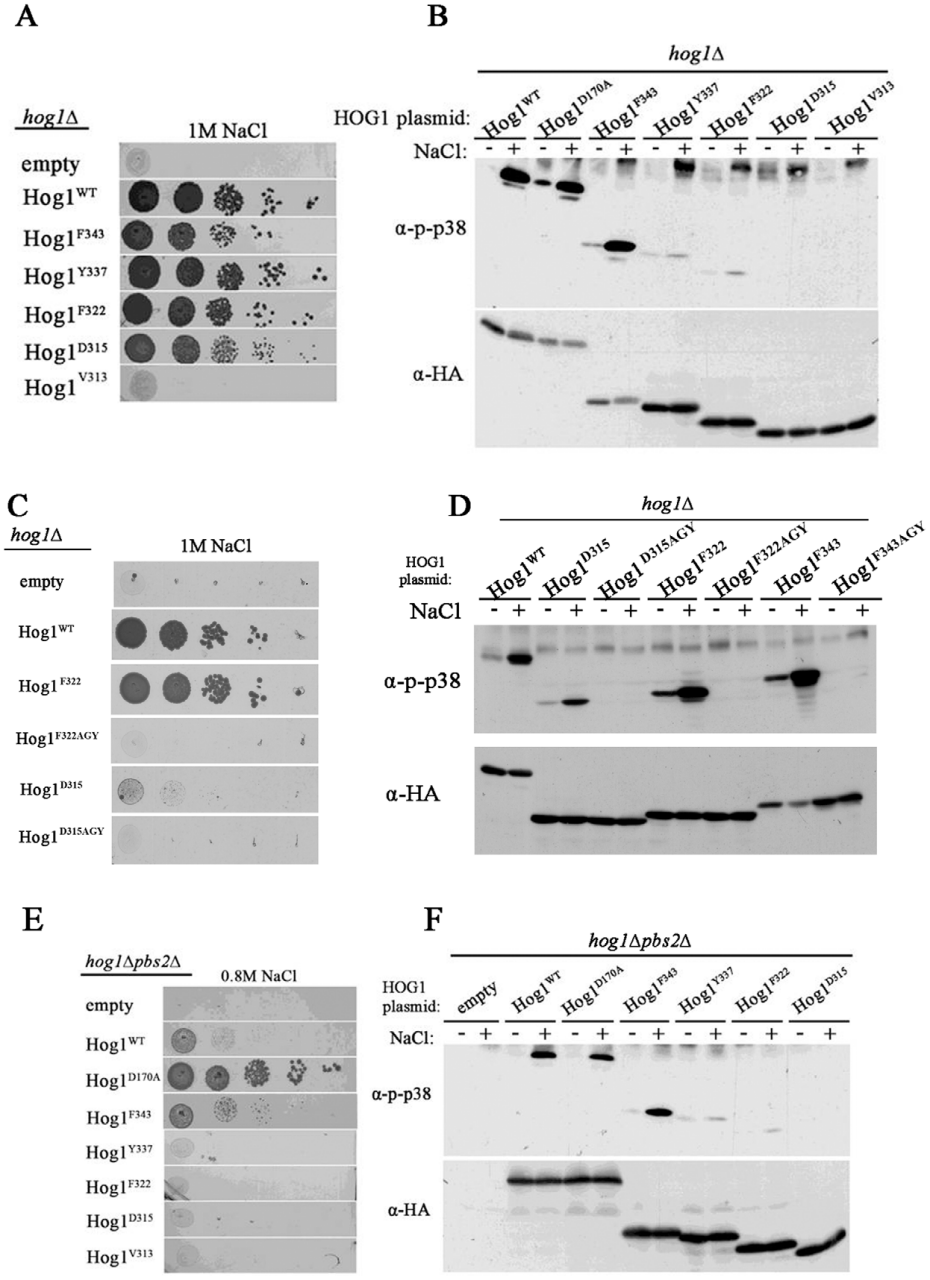
Hog1 molecules truncated up to D315 are activated by Pbs2, but molecules shorter than Y337 are not autoactivated. **A.** *hog1*Δ cells harboring either an empty vector (negative control) or vectors expressing either wild type Hog1 (Hog1^WT^), or the truncated proteins Hog1^F343^, Hog1^Y337^, Hog1^F322^, Hog1^D315^ and Hog1^V313^ were plated in five dilutions (starting from 200,000 cells) on plates containing YPD supplemented with 1 M NaCl. All strains were also plated on medium with no NaCl and all grew equally well on these plates (not shown). **B.** Phosphorylation levels of Hog1^WT^, intrinsically active Hog1 variant (Hog1^D170A^) and truncated Hog1 proteins Hog1^F343^, Hog1^Y337^ Hog1^F322^, Hog1^D315^ and Hog1^V313^ were tested when expressed in *hog1*Δ cells, before (−) and 10 minutes after (+) exposure to 1 M NaCl. 20 μg of protein lysates were loaded on each lane. **C.** *hog1*Δ cells expressing the indicated truncated proteins, carrying either Thr174, or Ala174, were plated in five dilutions (starting from 10,000 cells) on plates containing YPD supplemented with 1 M NaCl. All strains were also plated on medium with no NaCl and all grew equally well on these plates (not shown). **D.** Phosphorylation levels of the indicated Hog1 proteins, expressed in *hog1*Δ cells were tested as described in panel B. 30 μg of protein lysates were loaded on each lane. **E.** *hog1*Δ*pbs2*Δ cells harboring same plasmids described in panel A were plated in five dilutions, starting from 100,000 on plates containing YPD supplemented with 0.8 M NaCl. Growth of cells expressing the intrinsically active Hog1 variant (Hog1^D170A^) served as a positive growth control. All strains were also plated on medium with no NaCl and all grew equally well on these plates (not shown). **F.** Phosphorylation levels of the indicated Hog1 proteins were tested as described in panel B, but when expressed in *hog1*Δ*pbs2*Δ cells. 20 μg of protein lysates were loaded on each lane.

### Autophosphorylation of Hog1 is Mediated via a Domain that is also Important for Pbs2-Dependent Hog1’s Activation

The results above identified Hog1’s C-terminus (residues F343–Q435) as a domain that possesses some inhibitory properties on the protein’s Pbs2-independent activation. To assess if we identified the entire inhibitory domain or perhaps further deletions would increase spontaneous phosphorylation and improve growth of *pbs2*Δ cells under osmostress, we created 4 additional truncations: Hog1^Y337^, Hog1^F322^, Hog1^D315^ and Hog1^V313^ (Fig. 2, black arrows).

**Figure 5:**
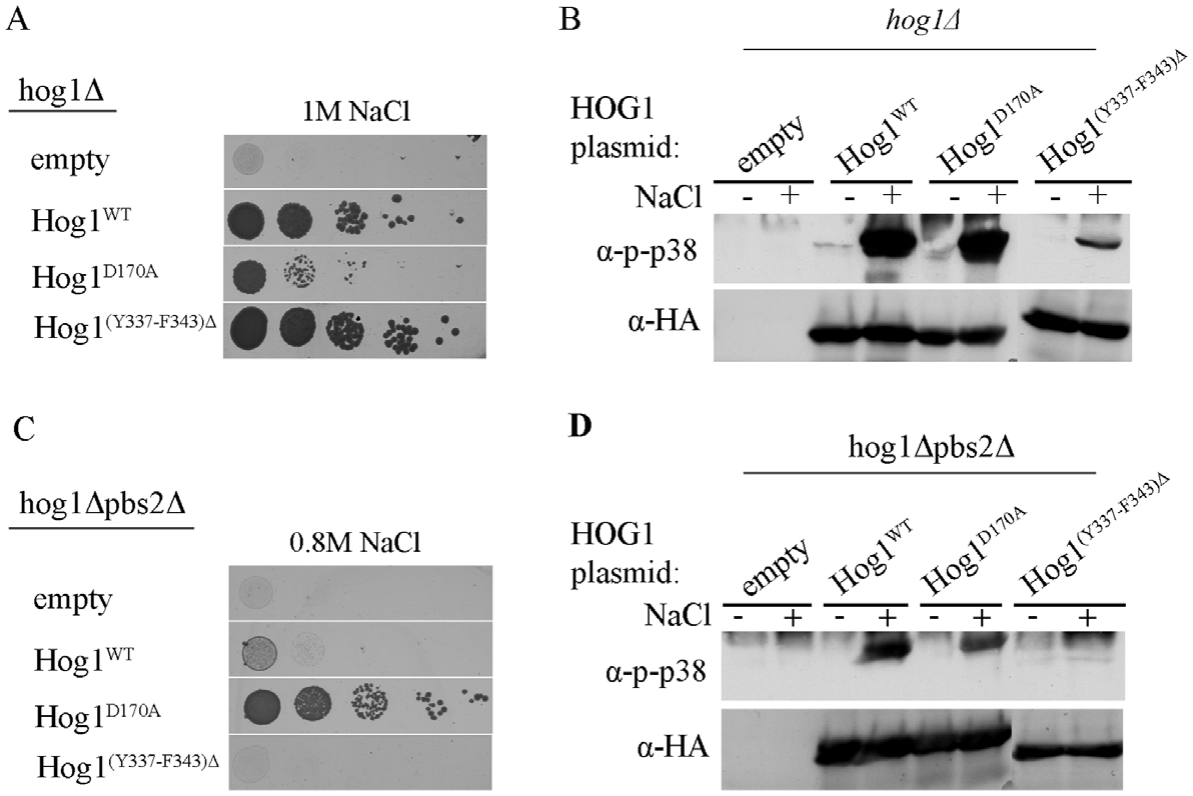
The region between Y337 and F343 is crucial for Pbs2-independent phosphorylation and activation of Hog1, but is dispensable for Pbs2-dependent Hog1’s biological activity. A. *hog1*Δ cells harboring either an empty vector (negative growth control), a vector expressing intrinsically active Hog1^D170A^, vectors expressing Hog1^WT^, or a vector expressing Hog1 missing the amino acids between Y337 and F343 (Hog1^(Y337–F343)Δ^) were plated on plates containing YPD supplemented with 1 M NaCl. All strains were also plated on medium with no NaCl and all grew equally well on these plates (not shown). B. Phosphorylation levels of Hog1^WT^, Hog1^D170A^ and Hog1^(Y337–F343)Δ^ were tested when expressed in *hog1*Δ cell. C. *hog1*Δ*pbs2*Δ cells harboring same plasmids as in A were plated on plates containing YPD supplemented with 0.8 M NaCl. *hog1*Δ*pbs2*Δ cells expressing the intrinsically active Hog1^D170A^ served as a positive control. All strains were also plated on medium with no NaCl and all grew equally well on these plates (not shown). D. Phosphorylation levels of Hog1^WT^, Hog1^D170A^ and Hog1^(Y337–F343)Δ^ were tested when expressed in *hog1*Δ*pbs2*Δ cells.

**Figure 6:**
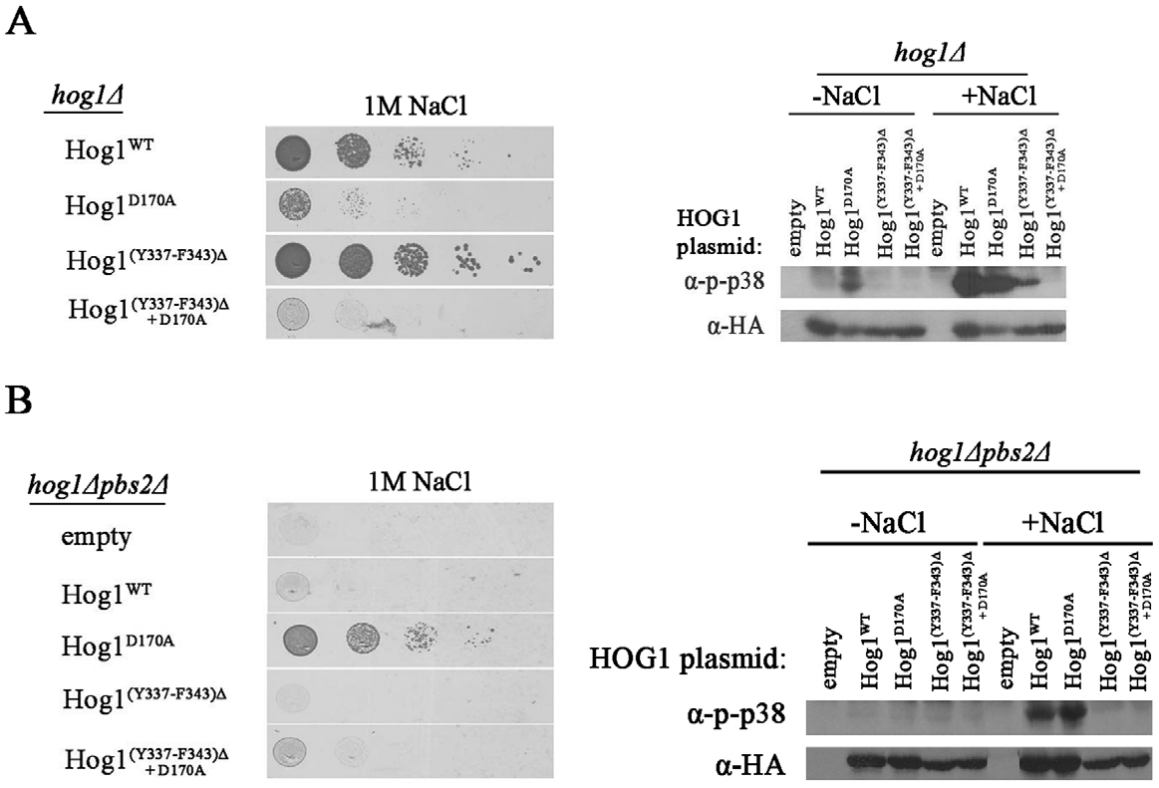
The region between F343 and Y337 is vital for the Hog1^D170A^ protein. **A.** *hog1*Δ cells harboring vectors expressing either Hog1^WT^, Hog1^D170A^, Hog1^(Y337–F343)Δ^, or Hog1^(Y337–343)Δ+D170A^ were plated in five dilutions on plates containing YPD supplemented with 1 M NaCl (left panel). All strains were also plated on medium with no NaCl and all grew equally well on these plates (not shown). Phosphorylation levels of Hog1^WT^, Hog1^D170A^, Hog1^(Y337–F343)Δ^, or Hog1^(Y337–343)Δ+D170A^ expressed in *hog1*Δ cells were tested in cells exposed or not exposed to NaCl (right panel). **B.** *hog1*Δ*pbs2*Δ cells harboring either an empty vector, or vectors expressing either Hog1^WT^, Hog1^D170A^, Hog1^(Y337–F343)Δ^, or Hog1^(Y337–343)Δ+D170A^ were plated in five dilutions on plates containing YPD supplemented with 1 M NaCl (left panel). All strains were also plated on medium with no NaCl and all grew equally well on these plates (not shown). Phosphorylation levels of Hog1^WT^, Hog1^D170A^, Hog1^(Y337–F343)Δ^, or Hog1^(Y337–343)Δ+D170A^ expressed in *hog1*Δ*pbs2*Δ cells were tested in cells exposed or not exposed to NaCl (right panel).

Expression of the new truncated Hog1 proteins in a *hog1*Δ strain revealed that all proteins, with the exception of Hog1^V313^, were able to support proliferation of these cells under high osmotic pressure (1 M NaCl; Fig. 4A). This result suggests that the region upstream to Asp315 is crucial for Hog1’s biological activity when Pbs2 is present. The entire downstream sequence, from Ala316 to Gln435 is dispensable for Hog1's capability to support growth under osmostress. Namely, the entire PBD-2 domain could be removed without affecting Hog1’s biological activity, but if, in addition to removing the PBD-2 domain, the CD domain is modified (shortened by just two residues), then the protein is not functional. Although Hog1^Y337^, Hog1^F322^ and Hog1^D315^ support growth under osmostress (Fig. 4A) their phosphorylation levels are reduced (Fig. 4B) suggesting that the domain N-terminally to F343 is important for efficient Pbs2-mediated phosphorylation. The low phosphorylation levels of the truncated proteins, mainly that of Hog1^D315^, may suggest that phosphorylation is not critical for their capability to allow growth under osmostress. However, when Thr174 of these truncated proteins was mutated to Ala they lost their ability to support growth under osmostress (Fig. 4C and 4D).

**Figure 7:**
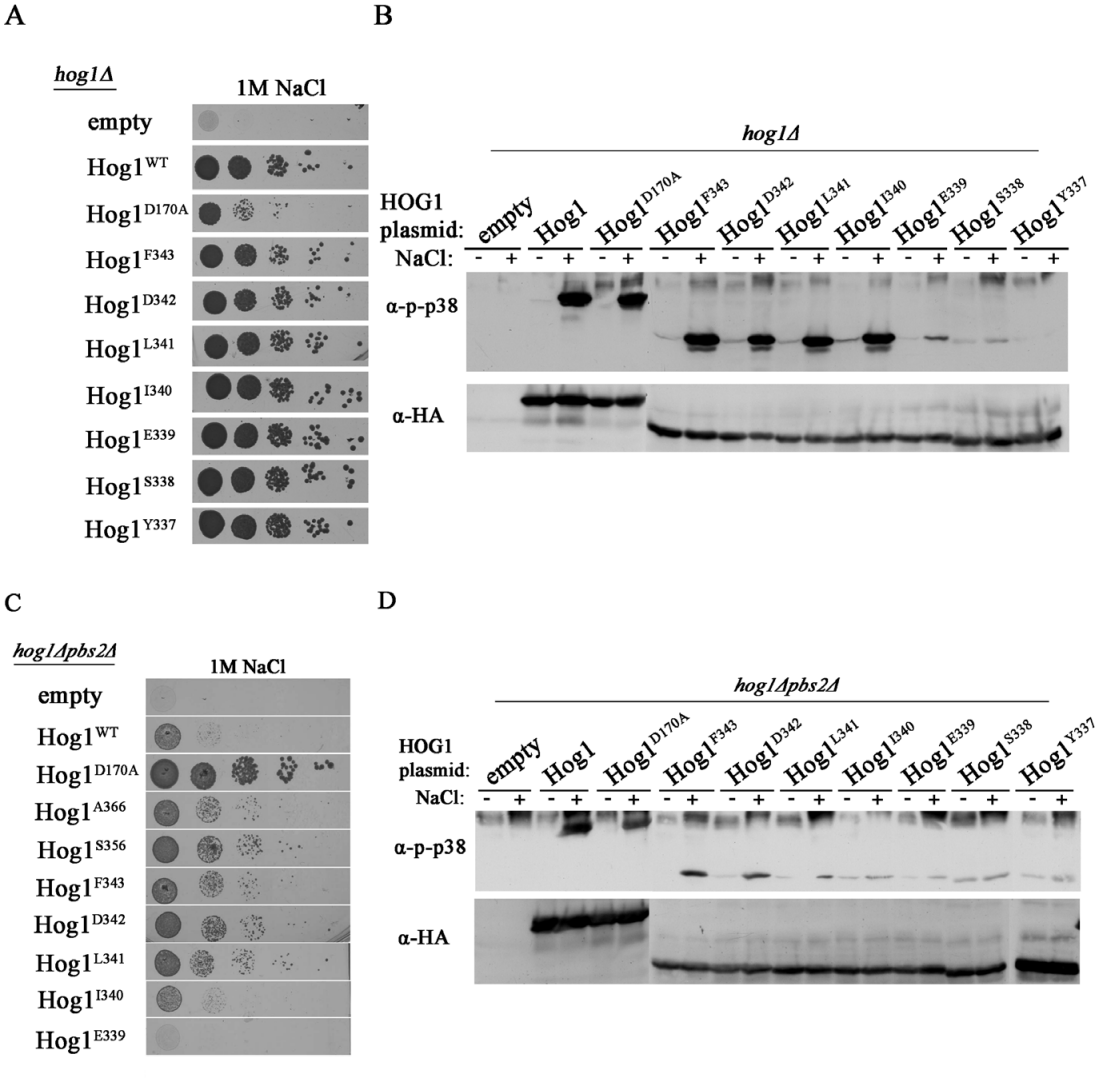
Hog1 proteins carrying serial deletions between F343 and Y337 are phosphorylated and biologically active in the presence of Pbs2. But, Hog1 proteins shorter than L341 are not activated in a Pbs2-independent manner. A. *hog1*Δ cells harboring either an empty vector (negative growth control), vector expressing either the intrinsically active variant Hog1^D170A^, or vectors expressing Hog1^WT^, Hog1^F343^, Hog1^D342^, Hog1^L341^, Hog1^I340^, Hog1^E339^, Hog1^S338^ or Hog1^Y337^ were plated in five dilutions on plates containing YPD supplemented with 1 M NaCl. All strains were also plated on medium with no NaCl and all grew equally well on these plates (not shown). B. Phosphorylation levels of Hog1^WT^, Hog1^F343^, Hog1^D342^, Hog1^L341^, Hog1^I340^, Hog1^E339^, Hog1^S338^ and Hog1^Y337^ were tested when expressed in *hog1*Δ cells. C . *hog1*Δ*pbs2*Δ cells harboring either an empty vector (negative control), a vector expressing intrinsically active variant Hog1^D170A^ (positive control), or vectors expressing either Hog1^WT^, Hog1^A366^, Hog1^S356^, Hog1^F343^, Hog1^D342^, Hog1^L341^, Hog1^I340^ or Hog1^E339^ were plated in five dilutions on plates containing YPD supplemented with 1 M NaCl. All strains grew equally well on media not supplemented with NaCl. D. Phosphorylation levels of Hog1^WT^, Hog1^F343^, Hog1^D342^, Hog1^L341^, Hog1^I340^, Hog1^E339^, Hog1^S338^ and Hog1^Y337^ were tested when expressed in *hog1*Δ*pbs2*Δ cells.

**Figure 8:**
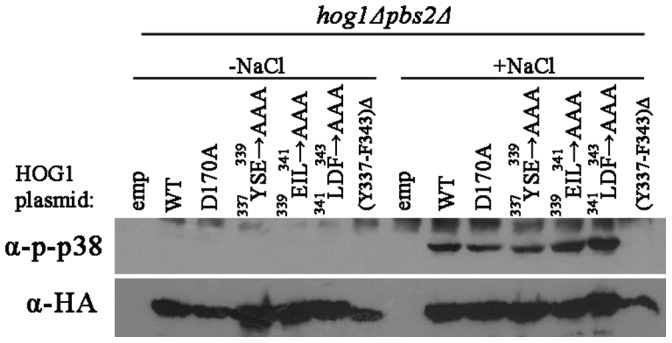
Systematic mutations of the residues between F343 to Y337 do not affect Pbs2-independent phosphorylation. Phosphorylation levels of Hog1 proteins carrying the indicated mutations were tested in *hog1*Δ*pbs2*Δ cells exposed (lanes 8–14) or not exposed (lanes 1–7) to 1 M NaCl.

**Figure 9:**
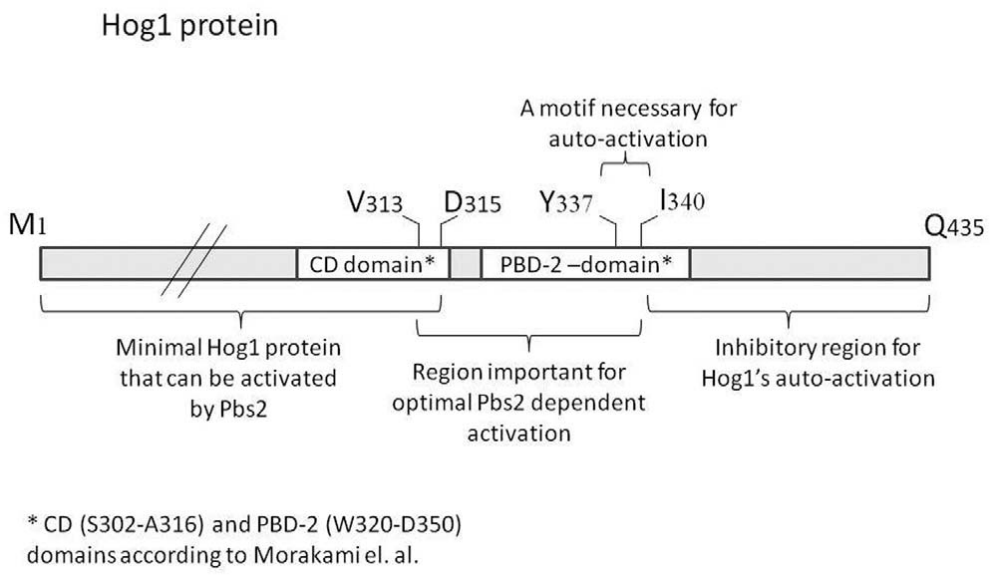
Schematic map of the Hog1 protein with the domains identified in this study and in the study of Murakami et al [**41**].

In principle, the above results are in agreement with those of Murakami *et al* [41] who mapped, the regions in Hog1 that are required for the interaction with Pbs2. They identified the region between amino acids W320 to D350 as crucial for this interaction and named it PBD-2 domain. Yet, as Pbs2 phosphorylates efficiently Hog1^F343^ and Hog1^F322^, the PBD-2 domain still probably covers a region further upstream of D350.

**Figure 10:**
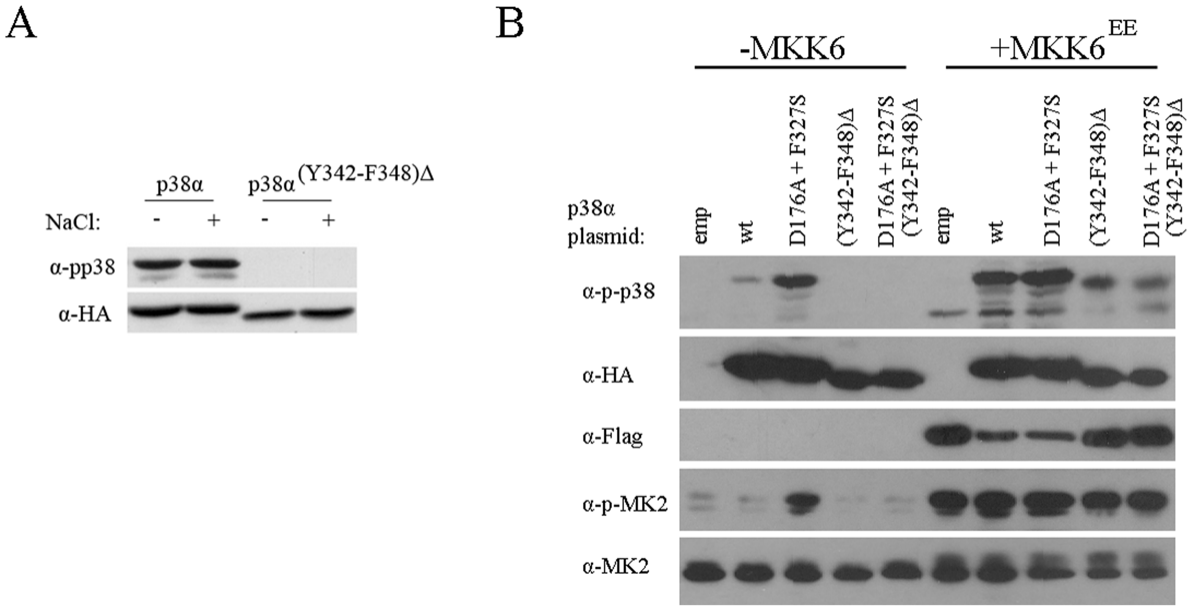
p38α^(Y342–F348)Δ^ is not autophosphorylated when expressed in yeast or in mammalian cells, but is phosphorylated by MKK6. A. Phosphorylation levels of p38α wild type (p38α) and p38α missing amino acids Y342–F348 (p38α^(Y342–F348)Δ^) were tested when expressed in *hog1*Δ*pbs2*Δ strain, before (−) and 10 minutes after (+) exposure to 1 M NaCl. B. Human embryonic kidney 293 cells were trasnfected with the indicated plasmids. Forty-eight hours after transfections cells lysates were prepared and analyzed by Western blots using the indicated antibodies. All p38 constructs are HA-tagged whereas MKK6^EE^ is flag-tagged. MK2 ( =  MAPKAPK2) is a substrate of p38α.

When expressed in *hog1*Δ*pbs2*Δ cells, proteins shorter than Hog1^F343^ did not allow growth under osmostress (Fig. 4E). Accordingly, proteins shorter than Hog1^F343^ manifested low phosphorylation when expressed in these cells (Fig. 4F). Namely, a Pbs2-independent autophosphorylation activity requires primarily the domain between amino acids F343 and Y337, which is part of the PBD-2 domain [41]. The fact that Pbs2-mediated activation and Pbs2-independent activation of Hog1 function via overlapping domains may suggest that the natural process of Hog1 activation combines these two mechanisms.

The above analysis allowed a distinction between three functional regions in the Hog1’s C-terminus domain: 1) an inhibitory domain of Hog1’s Pbs2-independent activation, includes the region from F343 down to the end of the protein (Q435). 2) A domain essential for autoactivation (Pbs2-independent) and also important for Pbs2-dependent phosphorylation (Y337–F343). 3) A domain (D315–Y337) that functions in the Pbs2-dependent phosphorylation of the protein, but is not essential for the biological activity of the Pbs2-Hog1 system (i. e., the system is still functional upon its removal). Only proteins shorter than Hog1^D315^ cannot be activated by Pbs2 to allow growth under osmostress.

The critical region for autoactivation and autophosphorylation of Hog1 seems to reside between Y337–F343, within the PBD-2 domain. To explicitly establish that these 7 amino acids are of importance, we created a protein lacking this region (Hog1^(Y337–F343)Δ^). When expressed in *hog1*Δ cells, the Hog1^(Y337–F343)Δ^ protein was biologically active just as Hog1^WT^, allowing yeast growth under osmotic stress (Fig. 5A), although its Pbs2-dependent phosphorylation levels were significantly reduced compared to Hog1^WT^ (Fig. 5B, lane 8). However, when expressed in *hog1*Δ*pbs2*Δ strain, Hog1^(Y337–F343)Δ^’S phosphorylation was barely detectable and did not allow any proliferation under osmostress (Fig. 5C, 5D). We also deleted the 7 amino acids region from the intrinsically active Hog1^D170A^ protein. This mutant can function biochemically and biologically independent of Pbs2, most probably because of an elevated autophosphorylation capability [22], [24], [39]. Removal of the 7 amino acids had a most dramatic effect on the Hog1^D170A^ protein, abolishing not only its unusual Pbs2-independent properties (Fig. 6B), but even its capability to be phosphorylated by Pbs2 and rescue *hog1*Δ cells (Fig. 6A).

These results indicate that the 7 amino acids between Y337 and F343 are crucial for stress-dependent autophosphorylation. As expected, as part of the PBD-2 domain [41], they are also important for efficient Pbs2-dependent Hog1 phosphorylation. They are totally dispensable however for Pbs2-dependent Hog1’s biological activity. In the context of the intrinsically active variant Hog1^D170A^, the 7 amino acids become even essential for its activity altogether.

We analyzed, therefore, the region between Y337 and F343 in more detail by creating the following series of truncated proteins: Hog1^F343^, Hog1^D342^, Hog1^L341^, Hog1^I340^, Hog1^E339^, Hog1^S338^ and Hog1^Y337^.

As expected, all proteins supported growth of *hog1*Δ cells under osmostress (Fig. 7A), although proteins shorter than Hog1^I340^ were phosphorylated to lower levels (Fig. 7B). When expressed in *hog1*Δ*pbs2*Δ strain, Hog1^I340^, Hog1^E339^, Hog1^S338^ and Hog1^Y337^ were not able to support proliferation under osmostress whereas Hog1^L341^ and longer allowed improved proliferation compared to Hog1^WT^ (Fig. 7A; Fig. 4E for Hog1^Y337^; for Hog1^S338^ data not shown). Accordingly, all proteins that allowed proliferation of *pbs2*Δ cells under osmostress, (Hog1^L341^ and longer; Fig. 7C) displayed increased phosphorylation in response to osmostress (Fig. 7D). Thus, L341 seems a critical residue for Pbs2-independent activation of Hog1 because truncation of the C-terminal region beyond it abolishes ability of the protein to rescue *pbs2*Δ cells (Fig. 7C). Notably however, mutating this residue to Ala did not affect autophosphorylation in *hog1*Δ*pbs2*Δ cells (Fig. 8; see below). In any case, the domain that inhibits autoactivation could be mapped downstream to L341 (Fig. 9).

We further mutated the amino acids residing between Y337 and F343, singly, or 3 together to Ala, in the context of the full-length protein. All mutated proteins were capable of rescuing *hog1*Δ cells from osmostress (data not shown) and all were phosphorylated in response to osmostress in *hog1*Δ*pbs2*Δ cells at levels similar to those of Hog1^WT^ (Fig. 8). Thus, although the presence of the 7 amino acids region in the Hog1 protein is of clear importance for autophosphorylation as well as for Pbs2-dependent phosphorylation (Fig. 5 and lane 14 in Fig. 8) its amino acids content seems to be less significant. Perhaps removing the 7 amino acids allows the C-terminal inhibitory domain to function more efficiently. Curiously, steady-state levels of the mutants carrying Ala residues seem to be higher than that of Hog1^WT^.

### Deletion of Amino Acids Y342 to F348 in the Human p38α Abolishes its Autophosphorylation Capability

The region identified to be essential for Hog1’s autoactivation, Y337–F343, is conserved in its mammalian ortholog p38α (Fig. 2) as well as in some other (but not all) MAP kinases [42]. To test the possibility that this sequence in p38α is functionally homologous and is essential for autophosphorylation, we deleted the region between Y342 and F348 from p38α (see Fig. 2), expressed the resulting protein in *hog1*Δ*pbs2*Δ yeast strain and tested whether p38α^(Y342–F348)Δ^ maintained autophosphorylation capability. p38α^WT^, which is constitutively autophosphorylated in yeast [27], [40], served as a control. The results (Fig. 10A) showed that p38α^(Y342–F348)Δ^ is not phosphorylated in yeast, suggesting that it lost its autophosphorylation ability.

We next tested the significance of this region for p38α’s activity in mammalian cells. For this purpose we also deleted the Y342–F348 region from the intrinsically active p38α^D176A+F327S^ protein. The p38α^D176A+F327S^ mutant manifests constitutive autophosphorylation and catalysis *in vitro* and *in vivo* (in mammalian cells; see refs. [24], [25] and lane 3 in Fig. 10B). We measured the phosphorylation levels of p38α^WT^, p38α^(Y342–F348)Δ^, p38α^D176A+F327S^ and p38α^D176A+F327S(Y342–F348)Δ^ when transiently expressed in HEK293 cells with or without co-expression of the active form of MKK6 (MKK6^EE^) which is a MAP2K of p38α. The results showed that in the absence of MKK6^EE^ p38α^D176A+F327S^ is phosphorylated and is capable of spontaneously phosphorylating the p38α substrate MAPKAPK2 (MK2) (lane 3 in Fig. 10B). The same protein lacking Y342–F348 lost spontaneous phosphorylation and activity (Fig. 10B, lane 5). p38α^WT^ is minimally phosphorylated when MKK6^EE^ is not co-expressed (Fig. 10B, lane 2) whereas p38α^(Y342–F348)Δ^ is not phosphorylated at all (lane 4). Both p38α^(Y342–F348)Δ^ and p38α^D176A+F327S(Y342–F348)Δ^ could be phosphorylated and activated by the active MKK6, but to a lower level than p38α^WT^ (Fig. 10B, compare lane 7 to 9 and lane 8 to 10 in the upper panel). These results show that just as in the case of Hog1, this region has a role both in the autophosphorylation of p38α and in efficient phosphorylation by the upstream MAP2K. This short region is crucial for autophosphorylation, but is not crucial for p38α (or Hog1) activation by the MAP2K.

## Discussion

Here we provided evidence that the yeast MAPK Hog1 could be regulated in a Pbs2-independent manner. Many previous studies did not observe Hog1 phosphorylation in *pbs2*Δ cells. This is probably because most studies used milder osmotic pressure (0.4 – 0.8 M NaCl) than we did (1 M NaCl). We, too, did not observe Hog1 phosphorylation in *pbs2*Δ cells exposed to a weaker pressure. We think that autophosphorylation of Hog1 might be biologically relevant because when sufficiently elevated, either by removing the C-terminal inhibitory region, or by insertion of activating mutations, it could relieve osmostress sensitivity of *pbs2*Δ cells. Our deletion analysis pointed at a 7 amino acids fragment in the C-terminus of Hog1 (Fig. 9), that if removed, autophosphorylation is abolished. This short domain may function via several possible mechanisms. It may serve for example as an interacting motif for an upstream regulator. It is interesting, in this context, that these 7 amino acids are part of the region that was reported to bind Pbs2 [41] and may serve therefore as a docking motif for several proteins (although they are not part of the common docking domain). Indeed, a Hog1 protein lacking this domain is weakly phophorylated by Pbs2 (Fig. 5B). On the other hand this protein is biologically functional, allowing growth in the presence of 1 M NaCl similar to Hog1^WT^ (Fig. 5A). The 7 amino acids may also function simply by controlling the adjacent inhibitory domain (Fig. 9). Their removal may bring the C-terminal inhibitory domain closer to sites important for activity. The involvement of the Y337–L341 region in Pbs2-dependent phosphorylation is not unexpected because it is part of a domain previously identified to interact with Pbs2 (PBD-2 domain). PBD-2 domain was mapped to residues 320–350 based on *in vitro* binding assays [41]. Our studies show that a Hog1 protein, missing about half of this region, Hog1^I340^ is efficiently phosphorylated by Pbs2, and even shorter proteins are still phophorylated to different levels. These shorter proteins, up to Hog1^D315^ may interact with Pbs2 via the CD domain that was mapped to S302–A316 [41]. Thus, the CD domain probably binds Pbs2 weakly and allows just partial phosphorylation of Hog1, but this seems sufficient for the biological effect. Although the 7 amino acids region seems to be extremely important for efficient Pbs2-mediated phosphorylation, mutatiing some of its residues to Ala, does not affect phosphorylation levels or biological activity (Fig. 8 and data not shown). This result may support the possible mechanism raised above that removal of the 7 amino acids region imposes conformational changes to control the adjacent C-terminal inhibitory domain and their exact sequence is not critical, as is, for activation. It may exist only for modulation of the C-terminal inhibitory domain.

Unexpectedly, the F343–Y337 region is critical for the functionality of the intrinsically active Hog1^D170A^ protein, even in *hog1*Δ cells (Fig. 6). We do not know how the 7 amino acids region may affect Asp/Ala170. Crystal structure of Hog1 is not available and in the crystal structure of p38α, D176 (D170’s ortholog) faces the solvent and does not interact with any other residue of the protein. In the structure of the intrinsically active p38α^D176A^ the phosphorylation lip is not apparent [43], [44]. Yet, the absolute dependence of Hog1^D170A^ on the 7 amino acids region serves as another indication for the importance of this region.

The F343–Y337 region is conserved in p38α at the levels of sequence and function. It is also conserved in other isoforms of p38 and in JNK, but is much less conserved in members of the ERK family. As many MAPKs may possess the capability of autophosphorylation [20], [21], [27], [45], it could be that in some of them (perhaps not in ERKs) it is mediated via this region. Curiously, Wilson et al., using unbiased bioinformatics tools, identified this region as part of a conserved motif, specific to MAP kinases, which resides within the non-conserved carboxy terminus [42].

Induction of Hog1^WT^ autophosphorylation in *pbs2*Δ cells does not support growth under osmostress. Yet, phosphorylation of Hog1^D170A^, Hog1^A366^, Hog1^S356^ and Hog^1F343^, significantly improves growth of *pbs2*Δ cells although they are phosphorylated to levels similar to those of Hog1^WT^. Perhaps there are particular functions, whose execution is not directly proportional to activation level, but rather to other properties that the mutants can acquire whereas the wild type protein cannot (e. g., subcellular localization, affinity to substrates). This notion is supported by the fact that some of the intrinsically active Hog1 mutants we described previously, such as Hog1^Y68H^ and Hog1^A314T^, manifest very low phosphorylation levels and catalytic activity in *pbs2*Δ cells, but are still capable of supporting growth of these cells under osmostress [22].

We do not know how the C-terminal inhibitory domain, Q435–L341, controls autophosphorylation. Its sequence does not contain any known binding site or a motif that can disclose its mechanism of action. Also, the sequence is specific to Hog1 and does not appear in any other proteins in the database. This domain may interfere with the ability of the nearby domain to impose autophosphorylation. It could also be that this domain is important for Hog1’s association with phosphatases. Several lines of evidence suggest that Hog1^WT^ undergoes some constitutive spontaneous phosphorylation reactions that are futile because they are instantly eliminated via a negative feedback machinery that probably activates phosphatases [46]. For example, deletion of genes encoding two phosphatases, *PTC1* and *PTP2* is lethal unless the *HOG1* pathway is inactivated [47]. The physiological significance of this constant futile cycle is not clear, but seems to exist in other MAPK pathways including *KSS1* and *FUS3* and in tyrosine kinases too [46], [48]. Mathematical models suggest that this basal signal transduction pathway is important for rapid and sensitive response of Hog1 [46].

What could be the role, if any, of the autophosphorylation process in the wild type cell? Several explanations may be suggested. For example, to maintain, as a sort of cellular memory, low levels of active Hog1 after stress is relieved and Pbs2 activity is reduced. Autophosphorylation may also function to activate Hog1 in some sub-cellular compartments in which Pbs2 does not exist. Finally, it could be that in the natural activation process Pbs2-depenednet phosphorylation is multiplied by autophosphorylation.

Many kinases have the ability of autophosphorylation which plays an important part in their activation [1]–[3]. As MAPKs are regulated by dual, MAP2K-mediated phosphorylation, and as their autophosphorylation activity was reported to be weak [44], [45], autophosphorylation and autoactivation are not considered an important element in their regulation. However, recent studies suggested that the autophosphorylation capability of MAPKs is in fact not weak, but should be evoked by specific regulators. p38α for example, is activated by pathways, which do not use the MAP3K-MAP2K cascade, but ignite autophosphorylation, in two different circumstances. One is when interacting with TAB1 in response to TGFβ and the other is in response to T-cell receptor activation, which leads to interaction between p38α and the kinase ZAP70 [20], [21]. Finally, when expressed in yeast, all ERK and p38 isoforms efficiently autophosphorylate [27]. Interestingly, JNKs seem not to autophophorylate (with the exception of the JNK2α2 isoform) [27], [49].

Thus, although the biological and physiological role of this type of activation is not clear yet, activation of MAPKs by autophosphorylation seems to be an evolutionarily conserved general phenomenon.
